# A Frameshift Mutation in *wcaJ* Associated with Phage Resistance in *Klebsiella pneumoniae*

**DOI:** 10.3390/microorganisms8030378

**Published:** 2020-03-07

**Authors:** Demeng Tan, Yiyuan Zhang, Jinhong Qin, Shuai Le, Jingmin Gu, Li-kuang Chen, Xiaokui Guo, Tongyu Zhu

**Affiliations:** 1 Shanghai Public Health Clinical Center, Fudan University, Shanghai 201508, China; 2 Institutes of Medical Sciences, Shanghai Jiao Tong University, Shanghai 200025, China

**Keywords:** phage therapy, phage-host interactions, phage-resistance, *Klebsiella pneumoniae*

## Abstract

Phage therapy is a potential and promising avenue for controlling the emergence and spread of multidrug-resistant (MDR) *Klebsiella pneumoniae*, however, the rapid development of anti-phage resistance has been identified as an obstacle to the development of phage therapy. Little is known about the mechanism employed by MDR *K. pneumoniae* strains and how they protect themselves from lytic phage predation in vitro and in vivo. In this study, comparative genomic analysis shows undecaprenyl-phosphate glucose-1-phosphate transferase (WcaJ), the initial enzyme catalyzing the biosynthesis of colanic acid, is necessary for the adsorption of phage 117 (*Podoviridae*) to the host strain Kp36 to complete its lytic life cycle. In-frame deletion of *wcaJ* alone was sufficient to provide phage 117 resistance in the Kp36 wild-type strain. Complementation assays demonstrated the susceptibility of phage 117, and the mucoid phenotype could be restored in the resistant strain Kp36-117R by expressing the wild-type version of *wcaJ*. Remarkably, we found that bacterial mobile genetic elements (*insA* and *insB*) block phage 117 infections by disrupting the coding region of *wcaJ*, thus preventing phage adsorption to its phage receptor. Further, we revealed that the wcaJ mutation likely occurred spontaneously rather than adapted by phage 117 predation under unfavorable environments. Taken together, our results address a crucial evolutionary question around the mechanisms of phage–host interactions, increasing our current understandings of anti-phage defense mechanisms in this important MDR pathogen.

## 1. Introduction

Bacteriophages are one of the most ubiquitous and abundant entities on earth, driving bacterial populations and evolutionary dynamics, as they exert strong selection pressures on susceptible populations [1]. Yet, bacteria have developed multiple defense mechanisms against phage infections and have stably coexisted among phages in nature [2]. Most bacterial phage resistance inevitably acquires genetic mutations, which is often associated with fitness trade-offs, however, non-mutational defense mechanisms that do not involve genetic changes are also widely distributed, allowing long-term coexistence between bacteria and predators [3,4]. Therefore, a comprehensive understanding of how phages interact with their corresponding host, as well as potential phage resistant mechanisms, is urgently needed, especially with regards to bacteria–phage coevolution in the context of human infections.

*Klebsiella pneumoniae* is a Gram-negative bacterium belonging to the family *Enterobacteriaceae* [5]. Recently, emerging carbapenem-resistant hypervirulent *K. pneumoniae* (hvKp) strains are becoming increasingly more resistant to antimicrobials through capture, accumulation, and dissemination of resistant genes [6]. This increased resistance poses a severe public crisis worldwide. There is, therefore, an increasing need to develop new therapeutic alternatives to combat infections of multidrug-resistant *K. pneumoniae*.

The application of phages as therapeutic or prophylactic treatments of *K. pneumoniae* in clinics has attracted increased attention as most of the *K. pneumoniae* clinical isolates are multidrug-resistant (MDR), thus restricting the available treatment options. Evidence demonstrating that phage therapy can successfully reduce morbidity and mortality in *K. pneumoniae* infections does exist [7,8]. However, successful application of these phage therapy is challenged by supplicated anti-phage resistant mechanisms that require a more detailed understanding and exploitation of phage–host interactions in *K. pneumoniae* [9].

Colanic acid (CA) has been previously shown to be the phage receptor for *Pectobacterium carotovorum* subsp. *carotovorum* phage pcc27 [10]. Tn5 transposon mutagenesis in genes associated with CA biosynthesis provided phage Pcc27 resistant to strain POP72 [10]. Previous studies showed that *wcaJ* determines colony morphology in *K. pneomoniae*, rendering mucoid phenotypes as smooth and dry [11]. In addition, Cai et al. [12] discussed that WcaJ was down-regulated at the level of translation, but not at the level of transcription or genetic mutation. They hypothesized that bacteria may use DNA methylation or histone modifications to prevent phage attacks, as has been previous reported in *Bacillus subtilis* [13]. There is no doubt that such a protection mechanism is certainly expected for in *K. pneomoniae*.

Little is known about the mechanisms of *wcaJ*-mediated resistance raised by the host in order to avoid predation by phages. Bacteria are able to combat evolutionary challenges (i.e., phage infection) by acquiring mobile genetic elements (MGEs) that move around within a genome, introducing random insertion and gene disruption. These mutations are likely associated with phage receptors, such as outer membrane protein, polysaccharide moieties, as well as bacterial capsules and appendages [14]. Recently, some MGEs acquired through horizontal gene transfer (HGT) were discovered to contribute towards protection against phage infections in *Lactobacillus johnsonii* [15]. Alternatively, *Pseudomonas aeruginosa* confers to phage resistance by losing a 219.6 kb genomic fragment. These studies emphasize that genetic diversity and genomic rearrangements play an important role in bacterial evolution and phage–host interactions. Thus, such mechanisms for preventing infection by lytic phages in *K. pneumoniae* may also be prevalent and bacteria could potentially benefit from the disruption of phage receptor genes.

In this study, we explored potential anti-phage defense mechanisms when bacterial strain Kp36 was exposed to lytic phage 117 during growth. Through comparative genomic analyses, we identified a novel anti-phage resistance mechanism through MGE-mediated gene (*wcaJ*) disruption to prevent the biosynthesis of the phage 117 receptor colanic acid. In the context of phage therapy, these results are highly relevant as they provide new insights into our current understanding of mechanisms of phage-resistance for this important pathogen in nosocomial infections.

## 2. Materials and Methods

### 2.1. Bacterial Strains, Bacteriophages, Plasmids, Oligos, and Growth Conditions

All bacteria, phages, and plasmids used in this study are listed in Table S1. The oligos used in this study were synthesized from Sangon Biotech (Shanghai, China) and are listed in Table S2. Cultures of *Escherichia coli* DH5α and *K. pneumoniae* were grown aerobically in LB (Miller) broth (L3522, Sigma-Aldrich, St. Louis, MO, USA) and/or agar (15 g/L, Sigma-Aldrich, St. Louis, MO, USA). When needed, antibiotics were added in the following concentrations: 100 µg/mL spectinomycin and 100 µg/mL apramycin for both *E. coli* and *K. pneumoniae*. The bacterial strain *K. pneumoniae* Kp36 was originally isolated from urine samples from patients with urinary tract infections in Shanghai, China, and phage 117, which infects Kp36, was isolated from sewage at Tzu Chi University, Hualien, Taiwan. Phage-resistant mutant Kp36-117R derived from *K. pneumoniae* Kp36 were selected for by confrontation with phage 117 and used as a host to proliferate phage 31 as described previously [6]. Both phages and their corresponding hosts have been recently characterized, paving the way for further molecular understanding of their interactions.

### 2.2. Phage Lysate Preparation

Single colonies of *K. pneumoniae* Kp36 and Kp36-117R were inoculated in LB culture and incubated at 37 °C with aeration. Subsequently, bacterial cultures were diluted in 1:1000 in 25 mL LB culture and grown to an optical density OD_600_ of 0.3 at 37 °C with aeration. Phage 117 and phage 31 were added at multiplicities of infection (MOI) of 0.1 and incubated at 37 °C without shaking to avoid bacterial aggregates formation for 6 h. In order to obtain high titer phage stock, the lysate was transferred to a sterile tube and centrifuged (12,000× *g*, 10 min, 4 °C). The supernatant was filtered through a 0.22 µm membrane (Millipore, Billerica, MA, USA), and the phage stock was stored at 4 °C. Phage lysate was enumerated by double-layer plaque assay.

### 2.3. Phage 117 Adsorption Rate Measurements

Overnight cultures of *K. pneumoniae* Kp36 and Kp36-117R were diluted 1000-fold in LB medium and grown at 37 °C with aeration until they reached OD_600_ = 0.6–0.9, corresponding to approximately 4.8 × 10^8^ CFU/mL. Phages were added at an MOI of 0.001. Aliquots were retrieved at two-minute intervals over 20 min. After adding the phages, aliquots were centrifuged at 16,000× *g* at 4 °C for two minutes and un-adsorbed phage particles of phage 117 in the supernatant were enumerated using the double agar overlay plaque assay. Adsorption rates were calculated based on the average of three independent experiments, with the slope of the function y = (ln(P0) − ln(P))/B0, where P0 and B0 are the numbers of phages and bacteria added at t = 0, respectively. Each experiment was performed in triplicate.

### 2.4. Bioinformatic Analysis of Phage-Resistant Mutant

Total genomic DNA of wild type Kp36 and phage-resistant mutant Kp36-117 were extracted from mid-log phase bacterial cultures according to the manufacturer’s protocol using Wizard Genomic DNA Purification Kit (Promega, Madison, WA, USA). The gDNA was sequenced at Sangon Biotech (Shanghai, China) using Illumina Hiseq platform (~1 Gbp/sample, paired-end). FastQC was used to assess quality control checks on raw reads. Low-quality reads and adapter sequences were trimmed by Trimmomatic. Following the Genome Analyzer Toolkit (GATK) best practices pipeline, we used the genomic mapping tool Burrows-Wheeler Aligner (BWA) to map low-divergent sequence to the reference genome of *K. pneumoniae*. Mutations, including base substitutions, deletions, and insertions, were detected by SAMtools, MarkDuplicates, and BEDTools. DNA frameshift mutations were further validated by PCR and sequencing. The genome sequences for *K. pneumoniae* Kp36 and Kp36-117R were deposited in GenBank under the accession numbers of CP047192 and GCF_009807065.1.

### 2.5. Construction and Characterization of Bacterial Mutants

In order to delete the complete open reading frame of *wcaJ* from the chromosome of *K. pneumoniae* Kp36, CRISPR-Cas9 spacer plasmid was constructed by cloning *wcaJ* sequence (AGACAAATACGATATGGTAT) into pSGKP-spe (Addgene no. 117234), as previously described [16]. For homologous recombination fragments, the upstream and downstream flanking regions of *wcaJ* were amplified, assembled in a second PCR, and cloned into CRISPR-Cas9 spacer plasmid pSGKP-spe-wcaJ. The resulting plasmids were extracted from *E. coli* DH5α and subsequently electroporated into a pCasKP-harboring strain *K. pneumoniae* Kp36. Desired mutants were sequence-verified using Sanger sequencing.

### 2.6. Complementation Assay

The intact *wcaJ* gene was amplified via PCR and cloned into the arabinose-inducible pBAD33 vector (kindly provide by Prof. Hongyu Ou) to generate pwcaJ plasmids [17]. The recombinant plasmids were first heat-shocked into *E. coli* DH5α and further electroporated into Kp36-117R. The Kp36-117R complementation strains were verified by PCR and sequenced using pBAD33 primers.

### 2.7. Spot Tests and Efficiency of Plaquing (EOP) Assays

The susceptibilities of the wild types Kp36, Kp36-117R, Kp36 Δ*wcaJ*, Kp36-117R pBAD33, and Kp36-117R pwcaJ strains were measured by spot assays to determine whether the *wcaJ* deletion had an effect on the outcome of phage infection. Phage lysates (117 and 31) were 100-fold serially diluted with SM buffer ranging from 10^0^ to 10^−6^. Aliquots of 2 µL phage dilutions were spotted on bacterial lawns mixed with 3 mL top agar (45 °C, 5 g/L) and 300 µL mid-log phase bacterial cultures. Spot test results were interpreted as ‘susceptible’ or ‘resistant’ after an overnight incubation at 37 °C. Additionally, plaque assays were conducted to enumerate the relative EOP of each individual strain by using the following formula: (the titer of target strain/the maximum titer of host strain). Each dilution was plated in duplicate to enhance accuracy.

### 2.8. Growth Curves of *K. Pneumoniae* Strains and Competition Assay

In an attempt to determine if the loss of gene *wcaJ* would affect bacterial growth in vitro, overnight cultures of *K. pneumoniae* strains Kp36, Kp36-117R, Kp36 Δ*wcaJ*, and Kp36-117R pwcaJ were diluted 1:1000 in LB broth and grown at 37 °C with aeration. The OD_600_ was measured at 1 h intervals over an 8 h incubation period. Linear growth rates and generation times of bacterial strains were calculated based on three biological replicates.

Additionally, we examined whether the loss of colanic acid would affect the outcome of potential competitive interactions between strains Kp36 and Kp36-117R. Briefly, mid-log phase cultures of *K. pneumoniae* strains Kp36 and Kp36-117R were back diluted 1000-fold in LB broth and mixed at the ratio of 1:1. Mixtures were incubated at 37 °C with aeration for 12 h, and subsequent subsamples were plated on LB plates. After 24 h of incubation at 37 °C, colonies were assessed by their clarity, being either ‘mucoid’ or ‘transparent’.

### 2.9. Luria–Delbrück Fluctuation Tests

Bacteria have the ability to resist phage predation by developing spontaneous mutations. Lamarck’s theories on evolution and inheritance suggest acquired immunity brought on by the environment, such as the prokaryotic CRISPR-Cas (clustered regularly interspaced short palindromic repeats–CRISPR-associated proteins) system of defense against mobile elements [18]. In contrast, Darwin proposed that mutants arising from spontaneous mutations happened to occur early in the development of a cellular population [18]. In 1943, Luria and Delbruck first conducted a series of experiments to address these conflicting hypotheses, suggesting that the phage-resistant mutations had a constant probability of occurring in each cell division [19].

In a first attempt to determine whether the *wcaJ* mutation arose in the absence of phage 117 selection, we conducted the Luria–Delbrück experiment, also known as the fluctuation test [20]. Overnight *K. pneumoniae* Kp36 was 100-fold diluted into LB cultures with 220 rpm agitation at 37 °C to reach the OD of 0.3 (ca. 10^8^ CFU/mL). Bacterial cultures were further diluted to a final concentration of 10^3^ CFU/mL. Aliquots of 10 mL bacterial dilutions were inoculated into one tube (group A), and aliquots of 1 mL of bacteria dilution was inoculated into 10 tubes (group B). All tubes were incubated at 37 °C without shaking until the ODs reached 0.6–0.8. Aliquots of 5 µL of bacterial cultures mixed with 200 µL phage 117 lysate (ca. 10^9^ PFU/mL) were plated onto LB agar plates. For group B, 10 technical replicates were sampled from the same tube. Non-mucoid bacterial colonies were enumerated after 24 h incubation at 37 °C.

## 3. Results

### 3.1. Morphological Differences between Wild-Type and Phage Resistant Mutants

**Figure 1:**
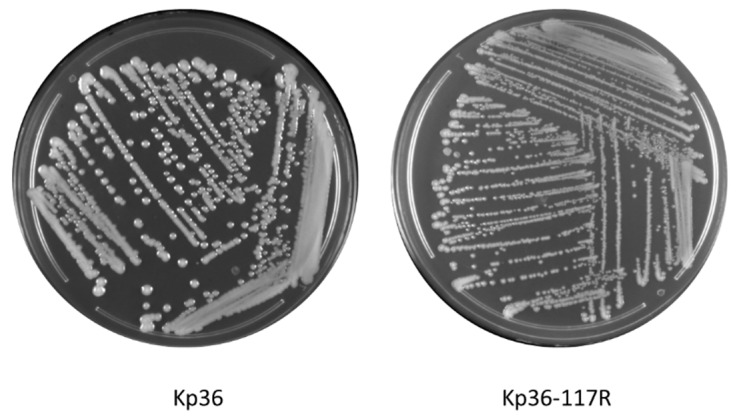
Morphology comparison between colonies of *K. pneumoniae* on parental strain Kp36 (mucoid, moist, and sticky) and phage 117-resistant mutant Kp36-117R (dry, rough, and transparent) on 1.5% LB agar.

One of the striking phenotypes observed in this study was rapid switching of colony morphology from mucoid to non-mucoid. In the absence of phage 117 infections, strain Kp36 displayed mucoid colony morphology of the wild-type, while under phage 117 treatment, strain Kp36-117R colonies were consistently dry, transparent, and relatively small (Figure 1). After re-streaking for four generations, this phage-resistant phenotype was heritage and persistent.

### 3.2. Phage Adsorption Assay Indicates Development of Resistance to Phage 117 during Adsorption

**Figure 2:**
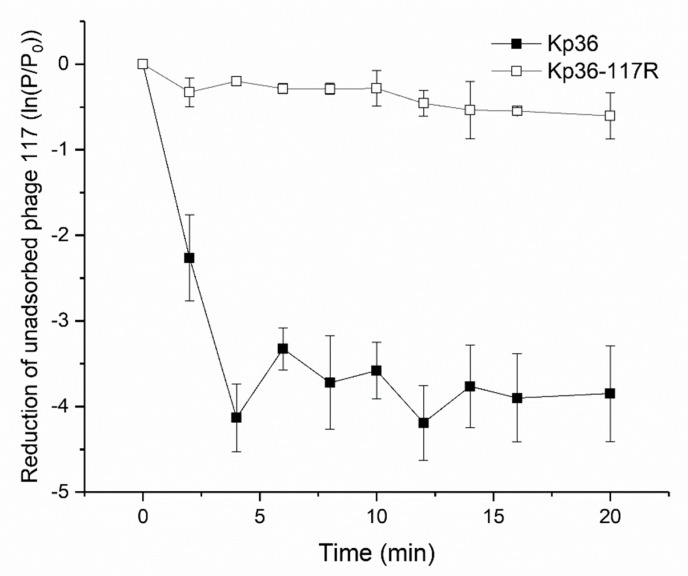
Phage-resistant mutant Kp36-117R displays different phage adsorption rates when compared with wild-type strain Kp36. Phage 117 was added to the mid-log phase cultures of strains Kp36 and Kp36-117R at an MOI of 0.001. Unabsorbed phage particles were harvested by centrifuge and counted by double-layer plaque assays. These experiments were performed in triplicate with error bars showing the variation between each experiment.

In order to address the question of how Kp36-117R becomes resistant to phage 117 mechanistically, we first quantified the rate of adsorption of phage 117 to strains Kp36 and Kp36-117R to investigate whether strain K36-117R resistance could be explained by preventing phage adsorption. The concentration of free phage 117 particles present in the culture medium represents the rate at which phage 117 particles unabsorbed to the host cells of Kp36 and Kp36-117R in the culture. The adsorption rates showed that ~80% phage particles accumulated in the supernatant of strain Kp36-117R, while the concentration of free phage 117 dropped dramatically by ~98% in strain Kp36 after four minutes (Figure 2). The adsorption rates of strains Kp36 and Kp36-117R were calculated as 1.66 × 10^−7^ ± 1.64 × 10^−8^ mL^−1^·s^−1^ and 3.65 × 10^−8^ ± 1.20 × 10^−8^ mL^−1^·s^−1^. Thus, the rate of adsorption of phage 117 was significantly lower than that observed for the strain Kp36-117R.

### 3.3. Characterization of the Phage Resistance Mechanism

**Figure 3:**
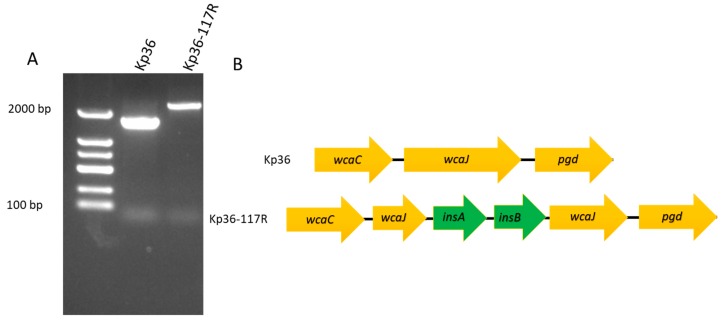
(**A**) Agarose gel electrophoresis of PCR products to verify the insertion of mobile genetic elements (*insA* and *insB*) in the phage resistant mutant Kp36-117R. Both PCR products, including wild-type strain Kp36, were confirmed by sequencing. (**B**) Schematic representation of *wcaJ* in *K. pneumoniae* Kp36 and Kp36-117R. Genes are represented as arrows.

Genome sequencing and comparative analysis of phage-resistant mutants and their parental strains have greatly improved our understanding regarding their anti-phage resistant mechanisms, phage–host interactions, as well as the overall biodiversity of their genomes. In order to identify the mutation(s) in Kp36-117R that might confer phage 117 resistance, as well as non-mucoid phenotype, the genomes of phage 117-resistant mutant strain Kp36-117R and the parental strain Kp36 were sequenced using Illumina Hiseq platform and analyzed. A total of 82 variations were detected between Kp36 and Kp36-117R. Out of the 82 variations, 13 were either insertions or deletions, with the rest of the 69 identified as single nucleotide polymorphisms (SNPs). These mutations were further evaluated by SnpEff to predict their variant effect, which was categorized into 51 intergenic regions, 22 synonymous variants, 8 missense variants, and 1 frameshift variant. Gene ontology (GO) enrichment analyses found that the outcome of the frameshift mutation completely altered the open reading frame of the *wcaJ* gene, having a high impact on the capsule polysaccharide biosynthetic process of strain Kp36. Moreover, both PCR and sequencing further verified the predicted *wcaJ* frameshifting mutation from the comparative genomic analysis above (Figure 3A). A 770 bp insertion sequence (IS1) derived from the parental strain Kp36 was detected between the *wcaJ* gene, which included two coding frames, *insA* and *insB*. As shown in Figure 3B, *insA* and *insB* were inserted into *wcaJ* in the following order (*wcaJ*, *insA*, *insB*, and *wcaJ*).

### 3.4. Complementation of Strain Kp36-117R with Wild-Type wcaJ Restores Ф117 Sensitivity

**Figure 4:**
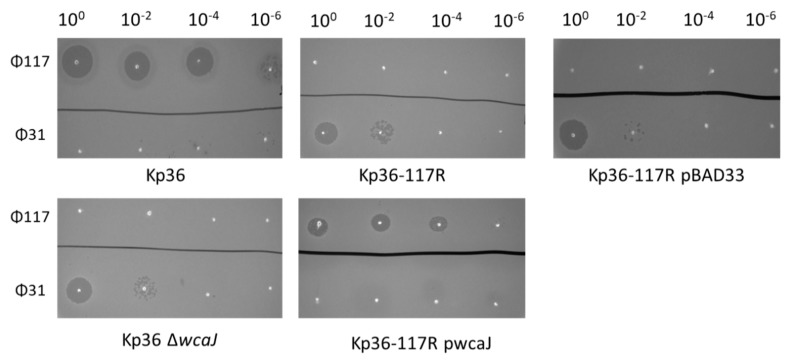
Spot test assay of phage 117 and phage 31 on the parental *K. pneumoniae* strain Kp36 and its derived mutants (Kp36-117R, Kp36 Δ*wcaJ*, Kp36 pBAD, and Kp36-117R pwcaJ).

In order to test whether complementation of strain Kp36-117R with wild-type *wcaJ* could restore phage 117 sensitivity and mucoid phenotypes, we amplified and cloned *K. pneumoniae* strain Kp36 *wcaJ* into plasmid vector pBAD33 (Figure 4). The resulting Kp36-117R pwcaJ mutant strain was verified by PCR and phage susceptibility. The results of spot tests and relative efficiency of plating (EOP) of phage 117 on strain Kp36-117R pwcaJ showed ~34% reduction as wild-type strain Kp36 (Table 1). Phage 31 was also included as a control as we demonstrated that *K. pneumoniae* strain Kp36 gained sensitivity to phage 31 once they became resistant to phage 117 (Figure 4). The EOP of phage 31 towards strains Kp36 Δ*wcaJ* and Kp36-117R pBAD ranged from ~0.70 to ~0.47 compared with its parental host strain Kp36-117R. In addition to phage sensitivity, the mucoid colony morphology phenotype was restored in the presence of plasmid pwcaJ in strain Kp36-117R pwcaJ.

### 3.5. The wcaJ Mutation Does Not Affect Growth Rate and Competition Assays

**Figure 5:**
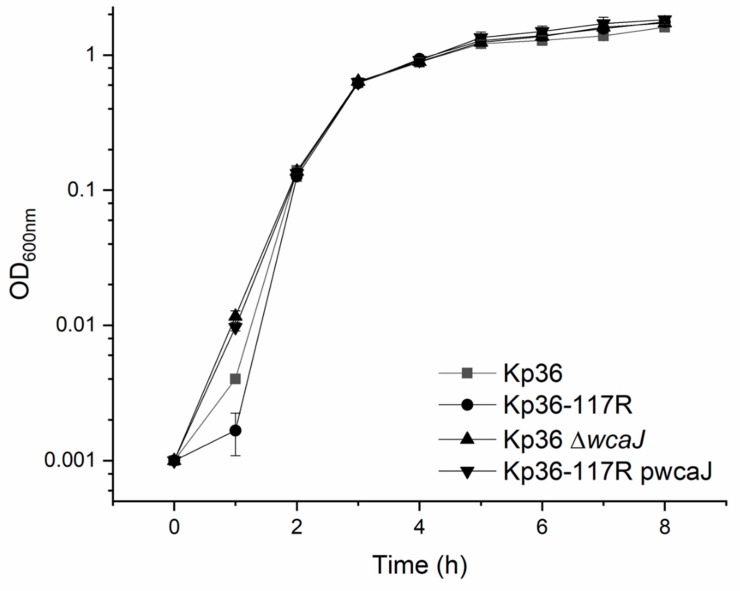
Growth curves of the wild-type and mutant of *K. pneumoniae* strains. No significant differences were observed among the parental *K. pneumoniae* strain Kp36 and its derivative mutants (Kp36-117R, Kp36 Δ*wcaJ*, and Kp36-117R pwcaJ) after 2 h. Optical densities (OD_600_) were measured at 1 h intervals over an 8 h period of incubation at 37 °C in LB broth with aeration. For strain Kp36-117R pwcaJ, media was supplemented with apramycin (100 μg/mL) and 0.4% L-arabinose. Error bars represent standard errors from the experiment performed in triplicate.

Most phage receptors are bacteria-encoded organelles located on cell surfaces, playing important functions such as mobility, nutrient transport, or providing integrity of the cell membrane. Therefore, bacteria would not benefit from subjecting their anti-phage defenses to receptor alteration or loss, and it should be advantageous to avoid maintaining constantly elevated anti-phage defenses. Thus, we evaluated whether *wcaj* alteration gives rise to a reduction in relative fitness in terms of growth rate and generation time. Surprisingly, there were no growth differences among all tested strains (Kp36, Kp36-117R, Kp36 Δ*wcaJ*, and Kp36-117R pwcaJ) after 2 h (Figure 5).

The physiological role of *wcaJ* had been previously well characterized, which is the initiating enzyme for colanic acid synthesis. To gain further insight into whether colanic acid plays an important role on ecological and evolutionary perspectives, we conducted competition assays between strains Kp36 and Kp36-117R. However, we were unable to detect any differences between the relative abundance of the *wcaJ* mutant Kp36-117R (6.27 × 10^8^ ± 2.05 × 10^8^ CFU/mL) when compared with the wild-type strain Kp36 (5.97 × 10^8^ ± 1.18 × 10^8^ CFU/mL) after an 8 h period of incubation in mixed culture. Thus, loss of the *wcaJ* gene does not directly affect the growth and competitive ability of strain Kp36-117R under LB medium conditions.

### 3.6. Non-Mucoid Phage Resistant Mutants are Unlikely Induced by Phage 117

**Figure 6:**
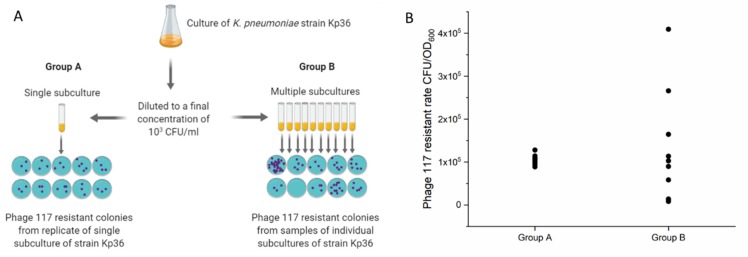
(**A**) Flow chart and schematic of the fluctuation experiment set-up. This figure was generated with the help from Biorender (https://biorender.com/). (**B**) The relative number of Phage 117 resistant colonies varies dramatically in group B, while the variation in group A is relatively small, indicating that phage 117-resistant mutants were likely aroused before phage infection.

A fluctuation test was performed to determine whether phage-resistant mutations arose randomly prior to phage 117 exposure (Figure 6A). As the colony morphology of phage-resistant mutant Kp36-117R changed dramatically from mucoid to non-mucoid, we used morphological difference to distinguish between wild-type (WT) and mutant. As shown in Figure 6B, samples from group A were taken from the same population and plated in the presence of phage 117, and the numbers of non-mucoid resistant colonies varied slightly. In contrast, samples from group B taken from different populations showed significant variations in the numbers of resistant colonies, ranging from 8 × 10^3^ CFU/OD to 4 × 10^5^ CFU/OD (Figure 6B). This variation represents the random fractions of resistant mutants within each population before phage 117 exposure, indicating that anti-phage mutation happened to accumulate early and successfully, and the populations would eventually be dominated by resistant mutants.

## 4. Discussion

In this study, we fully sequenced genomes of phage 117 resistant mutant Kp36-117R and wild-type Kp36 of *K. pneumoniae*, followed by further genetic and phenotypic characterization of the *wcaJ* frameshifting resistant mutant. Our results demonstrate that (i) *wcaJ* is linked to colanic acid synthesis, which affects the phage adsorption process; (ii) the acquired phage resistance was through the insertion of mobile genetic elements (*insA* and *insB*), causing a frameshifting mutation by *wcaJ*; and (iii) phage resistant mutants are very likely to have risen in the absence of phage 117 rather than being a response to phage adaptation.

Taken together, our data provide a deep insight into phage–host interactions in *K. pneumoniae* and strengthen our current understanding of how phage predation drives the evolution of this important nosocomial pathogen. Further studies on the mechanism of mobile genetic element-mediated frameshifting are needed, including identifying and evaluating the benefits and risks of using phages in clinical practices.

### 4.1. wcaJ Mutation Provides K. pneumoniae Strain Kp36 Phage Resistance by the Loss of Phage Receptor (Colanic Acid) Biosynthesis

Life cycles of lytic phages are a set of complex processes that involves and relies on a large number of steps. Bacteria have evolved multiple defense strategies to avoid, circumvent, subvert, and protect themselves against phage predation [2]. Adsorption is a key step in recognition between phage receptor-binding proteins and phage receptors on susceptible host cells [21]. Losses or changes to phage receptors in order to prevent phage adsorption can be the first step that bacterial cells exhibit in developing resistance to avoiding infection, as opposed to other resistant mechanisms that block phage DNA entry, replication, transcription, translation, or cell lysis [2].

In a first attempt, we conducted experiments to test whether phage 117-resistance of strain Kp36-117R would affect bacterial adsorption rates. We found that phage 117-resistant mutants showed a marked decrease in phage adsorption assays. Colanic acid has been previously identified and characterized as a phage receptor for *P. carotovorum* phage Pcc27 [10]. However, few studies addressed the genetic mechanisms employed by these phage resistant mutants in order to prevent phage adsorption. The capsular polysaccharide colanic acid, which has been implicated to protect bacteria in hostile environments, is organized by a highly conserved gene cluster among different *Enterobacteriaceae* strains [22]. In order to improve our understanding of the role and mechanism of colanic acid in phage susceptibility, we needed to investigate each gene locus. WcaJ is known as the initiating enzyme for colanic acid synthesis, transferring the glucose-1-phosphate moiety from undecaprenyl-phosphate glucose (UDP-Glc) onto the carrier lipid undecaprenyl phosphate [11]. Interestingly, Cai et al. [12] reported that WcaJ was significantly down-regulated in phage GH-K3 mediated *K. pneumoniae* K7R^R^ mutants. In their study, the potential mechanism was not likely to have involved genetic mutations, as they proposed that epigenetic modifications such as DNA methylation could be linked to the down-regulation of *wcaJ* transcription and expression levels. However, unlike previous findings, we mechanistically show that a frameshifting mutation in gene *wcaJ*, of which has been shown to be essential for colanic acid polysaccharide capsule synthesis, plays a crucial role for blocking the adsorption of phage 117. Our results add to the suite of known anti-phage mechanisms and their regulation of colanic acid synthesis in this important opportunistic pathogen and further emphasize that the complexity of phage–host interactions poses a challenge for future use of phages in clinical trials.

### 4.2. insA and insB Associated with a Frameshift Mutation in wcaJ

Comparative analysis of whole-genome sequencing (WGS) is a powerful bioinformatic tool that allows us to identify potential anti-phage defense mechanisms, while further improving our basic understanding of phage–host interactions on a molecular level [23]. In this study, WGS of strains Kp36 and Kp36-117R unraveled the evolutionary mechanism of phage 117 resistance in strain *K. pneumoniae* Kp36 under pressure by persistent phage predation. Specifically, a frameshifting event, which was mediated by coding frames *insA* and *insB*, is involved in production of the *wcaJ*-*insA*-*insB*-*wcaJ* gene cluster. The MGE-mediated frameshifting mutation was further accurately reflected by the resistant and susceptible phenotypes from in-frame deletions of *wcaJ* isogenic mutant (Kp36 Δ*wcaJ*) and complemented mutant (Kp36-117R pwcaJ), respectively.

In prokaryotes, the active transposable element, insertion sequence 1 (*insA* and *insB*), is wildly distributed in chromosomes and plasmids and involves various genomic rearrangements in certain bacteria belonging to the family *Enterobacteriaceae* [24]. The activities of transposons could change phage receptor properties, thereby promoting the maintenance of different susceptibilities to phages during infection. Using these strategies to switch between an “on” and “off” status, individual cells are able to evade phage attacks [25,26]. Unfortunately, the frameshifting mechanism is not resolved in our current study, especially as previous studies suggested that frameshifting events were likely to occur during translation of mRNA [27].

Colanic acid was previously demonstrated to be associated with virulence in *K. pneumoniae*, including evidence that colanic acid mutants cannot survive with macrophages. It was also found that *wcaJ* is essential for virulence in mice [12]. However, some studies do not support previous conclusions regarding the role of colanic acid in biofilm formation or virulence [28]. For instance, a study of uropathogenic *E. coli* and inert substrates demonstrated that colanic acid did not enhance bacterial adhesion, but rather blocked biofilm establishment, formation, and maturation [29]. Therefore, further studies are required to re-evaluate whether colanic acid affects virulence and associated fitness costs and benefits.

### 4.3. Phage 31 Infects Phage 117 Resistant Mutant

Microbial communities represent spatial physiological heterogeneity; even within a single phage–host interaction ecosystem, differential spatial and temporal gene expression occurs owing to phage predation and spontaneous host mutations [30,31]. In general, resistance is often associated with fitness costs (i.e., reduced abilities to take up nutrients or reduced competitive abilities); therefore, bacteria would benefit from subjecting their anti-phage defenses to a non-mutational defense mechanism. By creating a refuge to block a series of interactions between binding proteins of the phage and receptor on the bacterial cell surface, it offers a non-mutational defense mechanism to avoid maintaining consistently elevated resistant mechanisms [32,33]. Previous studies showed that *V. anguillarum* strain PF430-3 protected against phage KVP40 infections by increasing cell aggregation and biofilm formation, allowing coexistence rather than coevolution, promoting the stability of phage–host systems by reducing the risk of lytic phage attacks [4].

The recent discovery that phage 31 can form clear plaques on strain Kp36-117R containing a *wcaJ* frameshift mutation indicates that phage 31 does not utilize colanic acid as a receptor, and perhaps, the potential host receptor for phage 31 is likely blocked by colanic acid. As bacteria can produce capsular polysaccharides that act as physical barriers blocking antimicrobial compounds and phages, it is suggested to be a potential mechanism in play here [34,35]. In Gram-negative bacteria, many of the identified *Podoviridae* phage resistant genes are involved in polysaccharide synthesis [21]. Previous studies showed that colanic acid forms capsules that provide protection for bacteria living in biofilms. Once bacteria become resistant to phage 117, it will remove the colanic acid comprised exopolysaccharide from the bacterial surface, enabling other phages to adhere to outer membrane proteins and receptors. Indeed, further studies are needed to determine the receptor of phage 31 and to explore into the spatial localization between phage 117 receptor and phage 31 receptor.

## 5. Conclusions

Here, we demonstrate for the first time that phage resistance can be acquired through mobile genetic element-mediated mutations in gene *wcaJ*, which is critical for phage receptor colanic acid biosynthesis. The broad-host range spectrum of phage 117 is likely owing to the ubiquitous presence of capsular polysaccharide colanic acid, widely disturbed in most clinical Hypervirulent *K. pneumoniae* isolates as a receptor. However, it remains to be seen if additional mechanisms other than *wcaJ* mutations are responsible for phage 117 evading phage predation, specifically during clinical phage therapy treatments, and how the arm-raced phage–host interactions shape the epidemiology and virulence of multidrug-resistant *K. Pneumoniae* infections.

## Figures and Tables

**Table 1:** The sensitivities of each strain to phage 117 and phage 31 were shown by calculating the efficiency of plating (EOP) on *K. pneumoniae* strain Kp36 and its derived mutants (Kp36-117R, Kp36 Δ*wcaJ*, Kp36-117R pBAD, and Kp36-117R pwcaJ) and their plaque morphologies. WT, wild-type.

	Relative Efficiency of Plating		
*Klebsiella pneumoniae* strains	Phage 117 (*Podoviridae*)	Phage 31 (*Podoviridae*)	Plaque morphology
Kp36 (WT)	1	0	Clear
Kp36-117R (phage 117-resistant mutant)	0	1	Clear
Kp36 Δ*wcaJ* (*wcaJ* in-frame deletion)	0	0.70 ± 0.06	Clear
Kp36-117R pBAD (empty plasmid)	0	0.47 ± 0.07	Clear
Kp36-117R pwcaJ (Plasmid with complementary *wcaJ*)	0.66 ± 0.15	0	Turbid

## Supplementary Materials

Supplementary materials can be found at https://www.mdpi.com/2076-2607/8/3/378/s1.

## References

[1] Suttle C.A. (2005). Viruses in the sea. Nature.

[2] Labrie S.J., Samson J.E., Moineau S. (2010). Bacteriophage resistance mechanisms. Nat. Rev. Microbiol..

[3] Castillo D., Rørbo N., Jørgensen J., Lange J., Tan D., Kalatzis P.G., Svenningsen S.L., Middelboe M. (2019). Phage defense mechanisms and their genomic and phenotypic implications in the fish pathogen Vibrio anguillarum. FEMS Microbiol. Ecol..

[4] Tan D., Svenningsen S.L., Middelboe M. (2015). Quorum sensing determines the choice of antiphage defense strategy in Vibrio anguillarum. MBio.

[5] Podschun R., Ullmann U. (1998). Klebsiella spp. as nosocomial pathogens: Epidemiology, taxonomy, typing methods, and pathogenicity factors. Clin. Microbiol. Rev..

[6] Tan D., Zhang Y., Cheng M., Le S., Gu J., Bao J., Qin J., Guo X., Zhu T. (2019). Characterization of Klebsiella pneumoniae ST11 Isolates and Their Interactions with Lytic Phages. Viruses.

[7] Hung C.-H., Kuo C.-F., Wang C.-H., Wu C.-M., Tsao N. (2011). Experimental phage therapy in treating Klebsiella pneumoniae-mediated liver abscesses and bacteremia in mice. Antimicrob. Agents Chemother..

[8] Corbellino M., Kieffer N., Kutateladze M., Balarjishvili N., Leshkasheli L., Askilashvili L., Tsertsvadze G., Rimoldi S.G., Nizharadze D., Hoyle N. (2019). Eradication of a Multidrug-Resistant, Carbapenemase-Producing Klebsiella pneumoniae Isolate Following Oral and Intra-rectal Therapy With a Custom Made, Lytic Bacteriophage Preparation. Clin. Infect. Dis..

[9] Loc-Carrillo C., Abedon S.T. (2011). Pros and cons of phage therapy. Bacteriophage.

[10] Kim H., Kim M., Bai J., Lim J.-A., Heu S., Ryu S. (2019). Colanic Acid Is a Novel Phage Receptor of Pectobacterium carotovorum subsp. carotovorum Phage POP72. Front. Microbiol..

[11] Patel K.B., Toh E., Fernandez X.B., Hanuszkiewicz A., Hardy G.G., Brun Y.V., Bernards M.A., Valvano M.A. (2012). Functional characterization of UDP-glucose: Undecaprenyl-phosphate glucose-1-phosphate transferases of Escherichia coli and Caulobacter crescentus. J. Bacteriol..

[12] Cai R., Wang G., Le S., Wu M., Cheng M., Guo Z., Ji Y., Xi H., Zhao C., Wang X. (2019). Three capsular polysaccharide synthesis-related glucosyltransferases, GT-1, GT-2 and WcaJ, are associated with virulence and phage sensitivity of Klebsiella pneumoniae. Front. Microbiol..

[13] Goldfarb T., Sberro H., Weinstock E., Cohen O., Doron S., Charpak-Amikam Y., Afik S., Ofir G., Sorek R. (2015). BREX is a novel phage resistance system widespread in microbial genomes. Embo J..

[14] Hegstad K., Mikalsen T., Coque T., Werner G., Sundsfjord A. (2010). Mobile genetic elements and their contribution to the emergence of antimicrobial resistant Enterococcus faecalis and Enterococcus faecium. Clin. Microbiol. Infect..

[15] Guinane C.M., Kent R.M., Norberg S., Hill C., Fitzgerald G.F., Stanton C., Ross R.P. (2011). Host specific diversity in Lactobacillus johnsonii as evidenced by a major chromosomal inversion and phage resistance mechanisms. PLoS ONE.

[16] Wang Y., Wang S., Chen W., Song L., Zhang Y., Shen Z., Yu F., Li M., Ji Q. (2018). CRISPR-Cas9 and CRISPR-assisted cytidine deaminase enable precise and efficient genome editing in Klebsiella pneumoniae. Appl. Environ. Microbiol..

[17] Qian H., Yu H., Li P., Zhu E., Yao Q., Tai C., Deng Z., Gerdes K., He X., Gan J. (2019). Toxin–antitoxin operon kacAT of Klebsiella pneumoniae is regulated by conditional cooperativity via a W-shaped KacA–KacT complex. Nucleic Acids Res..

[18] Koonin E.V., Wolf Y.I. (2009). Is evolution Darwinian or/and Lamarckian?. Biol. Direct.

[19] Murray A. (2016). Salvador Luria and Max Delbrück on random mutation and fluctuation tests. Genetics.

[20] Luria S.E., Delbrück M. (1943). Mutations of bacteria from virus sensitivity to virus resistance. Genetics.

[21] Bertozzi Silva J., Storms Z., Sauvageau D. (2016). Host receptors for bacteriophage adsorption. FEMS Microbiol. Lett..

[22] Stevenson G., Andrianopoulos K., Hobbs M., Reeves P.R. (1996). Organization of the Escherichia coli K-12 gene cluster responsible for production of the extracellular polysaccharide colanic acid. J. Bacteriol..

[23] Ivanova N., Sorokin A., Anderson I., Galleron N., Candelon B., Kapatral V., Bhattacharyya A., Reznik G., Mikhailova N., Lapidus A. (2003). Genome sequence of Bacillus cereus and comparative analysis with Bacillus anthracis. Nature.

[24] Sekine Y., Ohtsubo E. (1989). Frameshifting is required for production of the transposase encoded by insertion sequence 1. Proc. Natl. Acad. Sci. USA.

[25] Zaleski P., Wojciechowski M., Piekarowicz A. (2005). The role of Dam methylation in phase variation of Haemophilus influenzae genes involved in defence against phage infection. Microbiology.

[26] Seed K.D., Faruque S.M., Mekalanos J.J., Calderwood S.B., Qadri F., Camilli A. (2012). Phase variable O antigen biosynthetic genes control expression of the major protective antigen and bacteriophage receptor in Vibrio cholerae O1. Plos Pathog..

[27] Jacks T., Madhani H.D., Masiarz F.R., Varmus H.E. (1988). Signals for ribosomal frameshifting in the Rous sarcoma virus gag-pol region. Cell.

[28] Ranjit D.K., Young K.D. (2016). Colanic acid intermediates prevent de novo shape recovery of Escherichia coli spheroplasts, calling into question biological roles previously attributed to colanic acid. J. Bacteriol..

[29] Hanna A., Berg M., Stout V., Razatos A. (2003). Role of capsular colanic acid in adhesion of uropathogenic Escherichia coli. Appl. Environ. Microbiol..

[30] Stewart P.S., Franklin M.J. (2008). Physiological heterogeneity in biofilms. Nat. Rev. Microbiol..

[31] Brockhurst M.A., Rainey P.B., Buckling A. (2004). The effect of spatial heterogeneity and parasites on the evolution of host diversity. Proc. R. Soc. Lond..

[32] Heilmann S., Sneppen K., Krishna S. (2012). Coexistence of phage and bacteria on the boundary of self-organized refuges. Proc. Natl. Acad. Sci. USA.

[33] Eriksen R.S., Svenningsen S.L., Sneppen K., Mitarai N. (2018). A growing microcolony can survive and support persistent propagation of virulent phages. Proc. Natl. Acad. Sci. USA.

[34] Scholl D., Adhya S., Merril C. (2005). Escherichia coli K1’s capsule is a barrier to bacteriophage T7. Appl. Environ. Microbiol..

[35] Schrag S., Mittler J. (1996). Host-parasite coexistence: The role of spatial refuges in stabilizing bacteria-phage interactions. Am. Nat..

