# Supplementary material for: A Frameshift Mutation in wcaJ Associated with Phage Resistance in Klebsiella pneumoniae
Source: Microorganisms. 2020 Mar 7;8(3):378. doi: 10.3390/microorganisms8030378 (PMC7142929; doi:10.3390/microorganisms8030378)
Supplement: Supplementary file 1 [file microorganisms-08-00378-s001.pdf]

microorganisms

MDPI

Table S1. Bacterial strains, bacteriophage and plasmids.

Strains, phage or plasmid	Genotype or relevant markers	Reference
Klebsiella pneumoniae		
Kp36	wild-type	[6]
Kp36-117R	phage 117 resistant mutant	[6]
Escherichia coli		
DH5α	Competent cells	
Phage		
phage 117	Genome 41,092 bp, *Podoviridae*	[6]
Phage 31	Genome 39,600 bp, *Podoviridae*	[6]
Plasmid		
pBAD33	*wcaJ* complementation assay	[17]
pSGKP-spe	*wcaJ* knockout	[16]
pCasKP	*wcaJ* knockout	[16]

Table S2. Primer list.

Name	Primers (5'-3')
wcaJ check	
*wcaJ*_check_F	CCCTGCCATGGTTATGGTGAG
*wcaJ*_check_R	CTCCTGTCAGGTGTGGTCAC
wcaJ deletion	
delta *wcaj*_1	TTTAAGCTTGATATCTTGACAGCTAGCTCAGTCCTAGGTATAATA -CTAGTAGACAAATACGATATGGTAtGTTTTAGAGCTAGAAATAGC
delta *wcaj*_2	TTTGAGCTCCACCGCGGTGGCGG
delta *wcaj*_3	TTTGAGCTCGGGATTATCGAACGGCAAATGGAG
delta *wcaj*_4	GTATCATAATTCGTACAATAGAAATCTCACCATAACCATGGCAGGG TATTG
delta *wcaj*_5	GGTTATGGTGAGATTTCTATTGTACGAATTATGATACTTAATCTTTTT TGTTGAGG
delta *wcaj*_6	TTTGAGCTCCCTTCGGCAGACAGCTCACGG
wcaJ complementation	
wcaJ comple_F	TTTTCTAGATAAGGAGGTATCATATGAAAACTTTCACGCATCGTGC
wcaJ comple_R	TTTAAGCTTTCAATATGCAGACTTATTAATAAACCC
